# The impact of dehydration on short-term postoperative complications in total knee arthroplasty

**DOI:** 10.1186/s12891-022-06118-7

**Published:** 2023-01-07

**Authors:** Brandon Lung, Kylie Callan, Maddison McLellan, Matthew Kim, Justin Yi, William McMaster, Steven Yang, David So

**Affiliations:** 1 Department of Orthopaedic Surgery, University of California, Irvine, 101 The City Dr S Pavilion 3, CA 92868 Orange, USA; 2 Department of Orthopaedic Surgery, Stony Brook University School of Medicine, NY Stony Brook, USA

**Keywords:** Knee arthroplasty, Postoperative complications, Dehydration status, BUN/Cr ration, NSQIP

## Abstract

**Background:**

As healthcare economics shifts towards outcomes-based bundled payment models, providers must understand the evolving dynamics of medical optimization and fluid resuscitation prior to elective surgery. Dehydration is an overlooked modifiable risk factor that should be optimized prior to elective total knee arthroplasty (TKA) to reduce postoperative complications and inpatient costs.

**Methods:**

All primary TKA from 2005 to 2019 were queried from the National Surgical Quality Improvement Program (NSQIP) database, and patients were compared based on dehydration status: Blood Urea Nitrogen Creatinine ratio (BUN/Cr) < 20 (non-dehydrated), 20 ≤ BUN/Cr ≤ 25 (moderately-dehydrated), 25 < BUN/Cr (severely-dehydrated). A sub-group analysis involving only elderly patients > 65 years and normalized gender-adjusted Cr values was also performed.

**Results:**

The analysis included 344,744 patients who underwent TKA. Adjusted multivariate logistic regression analysis showed that the severely dehydrated cohort had a greater risk of non-home discharge, postoperative transfusion, postoperative deep vein thrombosis (DVT), and increased length of stay (LOS) (all *p* < 0.01). Among the elderly, dehydrated patients had a greater risk of non-home discharge, progressive renal insufficiency, urinary tract infection (UTI), postoperative transfusion, and extended LOS (all *p* < 0.01).

**Conclusion:**

BUN/Cr > 20 is an important preoperative diagnostic tool to identify at-risk dehydrated patients. Providers should optimize dehydration to prevent complications, decrease costs, and improve discharge planning.

**Level of evidence:**

Level III; Retrospective Case-Control Design; Prognosis Study.

**Supplementary Information:**

The online version contains supplementary material available at 10.1186/s12891-022-06118-7.

## Introduction

As the demand for total knee arthroplasty (TKA) in an aging population increases, it is important for surgeons to medically optimize patients prior to surgery to decrease length of stay (LOS), reduce complications, and improve outcomes. With the emphasis on hospital quality metrics and value-based bundled payments, providers must understand the dynamics of medical optimization and fluid resuscitation before elective surgery. Preoperative hydration is a modifiable variable that can be addressed and monitored in clinic prior to surgery. Elderly patients undergoing joint replacement surgery have an increased risk of being dehydrated at baseline, and they are at an inherently high risk of requiring prolonged inpatient LOS and recovery due to underlying comorbidities and sensitivity to anesthesia [1]. While return to functional independence and ambulation are important goals following joint replacement, clinicians should be aware that dehydration may slow the rehabilitation process by causing orthostatic hypotension and fatigue [2].

Blood urea nitrogen (BUN) and creatinine are lab values commonly obtained prior to elective surgery to assess overall baseline renal function. A BUN to creatinine (BUN/Cr) ratio greater than 20 is a useful diagnostic screening tool to identify at-risk dehydrated patients who may be susceptible to acute inpatient complications. The BUN/Cr ratio has been validated as a sensitive marker for predicting dehydration, as previous studies have found physical symptoms, including low systolic blood pressure, dry mucous membranes, and sunken eyes, to have poor sensitivity for dehydration [3]. Risk stratification in the preoperative clinic using BUN/Cr may help surgeons identify potential patients who may need further discussion and expectation management of discharge planning. By improving orthostasis and circulatory function, earlier participation with physical therapy may speed functional recovery and improve discharge to home [2].

Previous studies in other surgical fields have analyzed the relationship between BUN/Cr and postoperative outcomes [4]. The simplicity and convenience of assessing BUN/Cr on routine preoperative clearance lab tests may prove cost-effective for early prevention of inpatient medical complications that may strain existing healthcare resources [5–7]. With an increasing demand for knee arthroplasty in a growing elderly population, it is important for providers to stratify patients who may need optimization and increased fluid intake prior to surgery. However, there is limited data to date in both orthopaedics and anesthesia. In this study, we use a national database from 2005 to 2019 to determine the correlation between the severity of preoperative BUN/Cr levels and postoperative outcomes to better provide clinical care and decision-making. Our hypothesis is that severely dehydrated patients undergoing TKA would have increased complications and LOS compared with adequately hydrated patients.

## Methods

All primary TKA from 2005 to 2019 with primary diagnosis of osteoarthritis were queried from the National Surgical Quality Improvement Program (NSQIP) database. The database was deidentified and exempt from approval by the Institution’s Institutional Review Board. Patient data was obtained from over 600 hospitals in the U.S. by certified healthcare workers through direct interviews, outpatient visits, and review of postoperative clinical notes [8]. It has been reported that the inter-reliability disagreement rate is estimated to be less than 2% [9]. The NSQIP database has been used for many other research studies relating to general orthopedics and joint arthroplasty [10–12].

Current Procedural Terminology code 27447 was used to identify all patients who underwent TKA from 2005 to 2019, and patients with Cr values > 1.3 mg/dL and a diagnosis of renal failure or dialysis were excluded to prevent poor kidney function as a confounding variable [13–15]. Patients without preoperative serum creatinine or BUN values were also excluded. The above exclusions resulted in a total of 344,744 cases for our statistical analysis. The study population was then divided into three different cohorts based on dehydration status: Bun/Cr < 20 (non-dehydrated), 20 ≤ Bun/C ≤ 25 (moderately-dehydrated), 25 < Bun/Cr (severely-dehydrated). A sub-group analysis involving only elderly patients > 65 years and normalized gender-adjusted Cr values (> 0.8 for males, and > 0.6 for females) was performed as low Cr values are often associated with malnutrition, advanced liver disease, and chronic kidney disease [16].

All statistical analyses for this study were conducted using Statistical Programs in Social Sciences Software version 26.0 (IBM Corporation, Armonk, NY, USA). Bivariate analysis was conducted to compare patient demographic characteristics, preoperative comorbidities, and procedural characteristics between levels of dehydration cohorts. A multivariate logistic regression analysis, which was adjusted for all notably associated variables such as patient demographics and preoperative comorbidities, was conducted to investigate the association between preoperative dehydration status and postoperative complications. Calculated odds ratios (OR) were reported in relation to the 95% confidence interval (CI). The level of statistical significance was set at *p* < 0.05.

## Results

### Patient demographics and distribution of dehydration levels

A total of 344,744 patients were included in the study, and 143,724 of those patients were 65 years or older. Roughly half of patients were not dehydrated according to the BUN/Cr ratio, and of those that were dehydrated, about half were moderately dehydrated and the other half were severely dehydrated (Table 1A). For the ≥65-year-old subgroup, about 60% of patients were not dehydrated, and of those that were dehydrated roughly 2/3 were moderately dehydrated and 1/3 were severely dehydrated (Table 1B).

**Table 1:** Comparison of dehydration levels for the full dataset and ≥ 65-year-old subgroup

**A. Full cohort BUN/Cr**			
		Count	Percent
Dehydration level	None (BUN/Cr < 20)	184,114	53.4%
	Moderate (25 > BUN/Cr ≥ 20)	90,210	26.2%
	Severe (BUN/Cr ≥ 25)	70,420	20.4%
	Total	344,744	100.0%
**B. ≥65 years subgroup BUN/Cr**			
		Count	Percent
Dehydration level	None (BUN/Cr < 20)	81,445	56.7%
	Moderate (25 > BUN/Cr ≥ 20)	39,217	27.3%
	Severe (BUN/Cr ≥ 25)	23,062	16.0%
	Total	143,724	100.0%

The raw count and percentage of patients in three bins of dehydration severity as defined by the pre-operative BUN/Cr ratio for the full dataset (Table 1A) and just the patients ≥65-years or older (Table 1B)

Before further analysis was possible, we determined which patient demographic variables co-varied with BUN/Cr ratio, and the distribution of other demographic parameters were calculated and compared for patients of all ages (Table 2). The mean age for severely dehydrated patients (68.5 ± 8.9 y [± std.]) was significantly different from both that of moderately dehydrated (67.6 ± 9.1 y, *p* < 0.001) and non-dehydrated patients (65.4 ± 9.6 y, *p* < 0.001), and the mean age for moderately dehydrated patients was different from non-dehydrated patients. (*p* < 0.001). Similarly, the mean body mass index (BMI) for non-dehydrated patients (33.2 ± 6.7) was different from both that of moderately (33.0 ± 6.8, *p* < 0.001) and severely dehydrated patients (32.8 ± 6.9, *p* < 0.001).

**Table 2:** Demographic information about patients undergoing Total Knee Arthroplasty

			Dehydration level		
			None	Moderate	Severe
Age	Mean	65.4		67.6	68.5
	St. dev.	9.6		9.1	8.9
	Count	184,055		90,163	70,359
Body Mass Index (BMI)	Mean	33.2		33.0	32.8
	St. dev.	6.7		6.8	6.9
	Count	183,653		89,950	70,206
Sex *p* < 0.001	Female	Count	83,859	28,703	13,626
		Percent	45.5%	31.8%	19.3%
	Male	Count	100,195	61,474	56,768
		Percent	54.4%	68.1%	80.6%
	Non-binary	Count	3	1	1
		Percent	0.002%	0.001%	0.001%
	Not reported	Count	57	32	25
		Percent	0.03%	0.04%	0.04%
Discharge destination *p* < 0.001	Home	Count	145,280	69,145	52,023
		Percent	78.9%	76.6%	73.9%
	Facility Which was Home	Count	863	410	351
		Percent	0.5%	0.5%	0.5%
	Multi-level Senior Community	Count	20	9	7
		Percent	0.01%	0.01%	0.01%
	Skilled Care, Not Home	Count	19,769	11,036	9798
		Percent	10.7%	12.2%	13.9%
	Rehab.	Count	11,126	6127	5664
		Percent	6.0%	6.8%	8.0%
	Separate Acute Care	Count	262	142	104
		Percent	0.1%	0.2%	0.1%
	Hospice	Count	6	2	4
		Percent	0.003%	0.002%	0.006%
	AMA	Count	20	7	1
		Percent	0.011%	0.008%	0.001%
	Expired	Count	41	27	17
		Percent	0.02%	0.03%	0.02%
	Unknown	Count	43	18	6
		Percent	0.02%	0.02%	0.01%
	Unskilled Facility Not Home	Count	157	102	74
		Percent	0.1%	0.1%	0.1%
	Not reported	Count	6527	3185	2371
		Percent	3.5%	3.5%	3.4%
Functional status prior to surgery *p* < 0.001	Independent	Count	181,109	88,755	69,180
		Percent	98.4%	98.4%	98.2%
	Partially Dependent	Count	1972	1005	880
		Percent	1.1%	1.1%	1.2%
	Totally Dependent	Count	52	32	38
		Percent	0.03%	0.04%	0.05%
	Unknown	Count	981	418	322
		Percent	0.5%	0.5%	0.5%
Anesthesia type *p* < 0.001	General	Count	87,777	40,191	30,633
		Percent	47.7%	44.6%	43.5%
	Monitored Anesthetisia Care (MAC)/Intravenous(IV) Sedation	Count	24,049	11,898	9192
		Percent	13.1%	13.2%	13.1%
	Regional	Count	3026	1451	1183
		Percent	1.6%	1.6%	1.7%
	Spinal	Count	67,337	35,709	28,691
		Percent	36.6%	39.6%	40.7%
	Epidural	Count	1626	848	631
		Percent	0.9%	0.9%	0.9%
	Local	Count	52	28	23
		Percent	0.03%	0.03%	0.03%
	Other	Count	171	51	41
		Percent	0.1%	0.1%	0.1%
	None	Count	46	23	17
		Percent	0.02%	0.03%	0.02%
	Unknown	Count	22	5	6
		Percent	0.01%	0.01%	0.01%
	Not reported	Count	8	6	3
		Percent	0.0%	0.0%	0.0%
Diabetes management *p* < 0.001	None	Count	152,605	74,217	57,826
		Percent	82.9%	82.3%	82.1%
	Non-insulin	Count	24,080	12,285	9600
		Percent	13.1%	13.6%	13.6%
	Insulin	Count	6938	3446	2763
		Percent	3.8%	3.8%	3.9%
	Oral	Count	491	262	231
		Percent	0.3%	0.3%	0.3%
History of dyspnea *p* < 0.001	No	Count	173,717	85,284	66,745
		Percent	94.4%	94.5%	94.8%
	At rest	Count	358	137	111
		Percent	0.2%	0.2%	0.2%
	Moderate	Count	10,039	4789	3564
		Percent	5.5%	5.3%	5.1%
Smoking history within 1 year *p* < 0.001	No	Count	164,847	84,467	66,684
		Percent	89.5%	93.6%	94.7%
	Yes	Count	19,267	5743	3736
		Percent	10.5%	6.4%	5.3%

Severely demographic and medical factors were considered from the dataset and displayed here. For age and BMI, means and standard deviations were calculated. For all others, counts and percentages were calculated. All counts are “valid” counts, meaning they were only tallied if the patient had a known value for the given variable. *P*-values on the left-hand side indicate the results of Chi-squared tests of independence

Gender was the only demographic variable in Table 2 to have a clear column-wise relationship upon inspection. To test the hypothesis that gender was related to the rate of dehydration, we used a full-factorial univariate general linear model (GLM) controlling for age and BMI to predict the BUN/Cr ratio. This revealed patients of non-female genders had 2.998–point increase in marginal mean BUN/Cr ratio (marginal mean difference: *p* < 0.001, 95% CI 2.950–3.047; GLM gender term: Type III sum of squares = 7.1 × 10^5^, F = 1.5 × 10^4^, *p* < 0.001), consistent with our findings in Table 2. In summary, age, BMI, and gender were identified as demographic variables to control for in further analysis.

### Rates of post-operative complications differs based on dehydration status

To determine how the level of dehydration affected the rate of complications we used a series of multivariate logistic regressions controlling for age, gender, BMI, pre-operative sodium, pre-operative albumin, and American Society of Anesthesiologists (ASA) class. For patients of all ages, it was found that the rate (assessed by odds ratio, OR) of non-home discharge (OR = 1.029, CI (95%) = 1.029–1.029, *p* = 0.031) and the rate of urinary tract infections (UTIs) (OR = 1.205, CI = 1.205–1.206, *p* = 0.002) were greater in moderately dehydrated patients compared to that of non-dehydrated patients. For all ages, severe dehydration was associated with greater rates of non-home discharge (OR = 1.103, CI = 1.103–1.103, *p* < 0.001), postoperative transfusion (OR = 1.171, CI = 1.170–1.171, *p* = 0.031), acquiring postoperative deep vein thromboses (DVTs) (OR 1.145, CI = 1.144–1.146, *p* = 0.039), and having a LOS > 2 days (OR = 1.069, CI = 1.069–1.069, *p* = 0.031) (all summarized in Table 3). No other significant associations were found.

**Table 3:** Comparison of complication rates following Total Knee Arthroplasty between Dehydration Status Cohorts

Complication	Moderately Dehydrated		Severely Dehydrated	
	odds ratio (*p* value)	95% CI	odds ratio (*p* value)	95% CI
Non-home discharge	1.029 (0.031*)	**1.029–1.029**	1.103 (< 0.001***)	**1.103–1.103**
Infection	0.980 (0.714)	0.980–0.981	0.901 (0.098)	0.901–0.092
Cardiac arrest or MI	0.982 (0.876)	0.981–0.984	0.988 (0.925)	0.987–0.990
Wound disruption	0.879 (0.291)	0.878–0.880	0.884 (0.368)	0.882–0.885
Pneumonia	0.947 (0.587)	0.946–0.948	1.100 (0.378)	1.099–1.102
Unplanned intubation	1.106 (0.485)	1.104–1.107	0.927 (0.650)	0.925–0.928
Pulmonary embolism	0.954 (0.532)	0.953–0.955	0.883 (0.141)	0.882–0.884
On ventilator > 48 h	1.098 (0.662)	1.096–1.101	0.839 (0.503	0.836–0.841
Progressive renal insufficiency	0.981 (0.920)	0.979–0.984	1.110 (0.607)	1.107–1.113
Acute renal failure	0.755 (0.325)	0.752–0.757	0.577 (0.123)	0.575–0.580
Urinary tract infection	1.205 (0.002**)	**1.205–1.206**	1.122 (0.088)	1.121–1.123
Stroke or CVA	1.047 (0.811)	1.044–1.049	1.095 (0.659)	1.092–1.097
Postoperative transfusion	1.003 (0.910)	1.003–1.004	1.171 (< 0.001***)	**1.170–1.171**
DVT	1.016 (0.793)	1.016–1.017	1.145 (0.039*)	**1.144–1.146**
Sepsis	0.996 (0.977)	0.995–0.998	1.033 (0.817)	1.032–1.035
Septic shock	1.282 (0.270)	1.278–1.285	0.848 (0.564)	0.845–0.851
C. diff.	0.823 (0.412)	0.821–0.826	0.637 (0.117)	0.635–0.639
Periprosthetic fracture	0.876 (0.257)	0.875–0.877	0.999 (0.996)	0.998–1.001
Length of stay > 2d	0.988 (0.292)	0.988–0.988	1.069 (< 0.001***)	**1.069–1.069**

For each of the above complications, individual multivariate binary logistic regression models were fit to predict the effect of dehydration on the rate of each complication. The model was controlled for the potential effects sex, age, BMI, pre-operative sodium, pre-operative albumin, and ASA class. The resulting model yielded odds ratios for moderate (25 > BUN/Cr ≥ 20) and severe (BUN/Cr ≥ 25) dehydration with respect to non-dehydrated patients. 95% confidence intervals are shown

In the subgroup of patients ≥65 years old, similar associations with complication rates were found. For those subgroup patients moderately dehydrated, the rate of non-home discharge (OR = 1.068, CI = 1.068–1.068, *p* < 0.001), UTIs (OR = 1.325, CI = 1.320–1.326, *p* < 0.001), and LOS > 2 d (OR = 1.035, CI = 1.035–1.035, *p* = 0.041) were all significantly elevated. For severely dehydrated patients, the rates of non-home discharge (OR = 1.151, CI = 1.151–1.152, *p* < 0.001), progressive renal insufficiency (OR = 1.675, CI = 1.669–1.680, *p* = 0.049), UTIs (OR = 1.231, CI = 1.229–1.232, *p* = 0.04), postoperative transfusion (OR = 1.296, CI = 1.296–1.297, *p* < 0.001), and LOS > 2 d (OR = 1.113, CI = 1.113–1.113, *p* < 0.001) were all significantly greater as well (all results summarized in Table 4). No other significant associations were found.

**Table 4:** Comparison of complication rates following Total Knee Arthroplasty between Dehydration Status Cohorts in elderly subgroup

Complication	Moderately Dehydrated		Severely Dehydrated	
	odds ratio (*p* value)	95% CI	odds ratio (*p* value)	95% CI
Non-home discharge	1.068 (< 0.001***)	**1.068–1.068**	1.151 (< 0.001***)	**1.151–1.152**
Infection	1.062 (0.447)	1.061–1.063	0.865 (0.169)	0.864–0.866
Cardiac arrest or MI	1.023 (0.877)	1.021–1.024	1.026 (0.882)	1.024–1.024
Wound disruption	1.150 (0.408)	1.148–1.152	0.757 (0.242)	0.755–0.759
Pneumonia	1.043 (0.760)	1.041–1.045	1.130 (0.442)	1.128–1.133
Unplanned intubation	1.042 (0.827)	1.040–1.045	1.102 (0.660)	1.099–1.105
Pulmonary embolism	1.001 (0.995)	0.999–1.002	1.023 (0.860)	1.021–1.024
On ventilator > 48 h	1.038 (0.891)	1.035–1.042	0.757 (0.454)	0.753–0.760
Progressive renal insufficiency	0.903 (0.703)	0.900–0.906	1.675 (0.049*)	**1.669–1.680**
Acute renal failure	0.782 (0.532)	0.778—0.785	0.825 (0.678)	0.820–0.829
Urinary tract infection	1.325 (< 0.001***)	**1.32–1.326**	1.231 (0.040*)	**1.229–1.232**
Stroke	1.020 (0.934)	1.017–1.023	1.075 (0.789)	1.072–1.079
Postoperative transfusion	1.069 (0.100)	1.069–1.070	1.296 (< 0.001***)	**1.296–1.297**
DVT	0.985 (0.866)	0.984–0.986	1.218 (0.054)	1.216–1.219
Sepsis	1.335 (0.083)	1.332–1.337	1.110 (0.626)	1.108–1.113
Septic shock	0.979 (0.940)	0.975–0.82	0.852 (0.657)	0.848–0.856
C. diff	0.535 (0.067)	0.535–0.533	0.712 (0.362)	0.708–0.715
Periprosthetic fracture	0.920 (0.619)	0.918–0.922	0.898 (0.598)	0.896–0.900
Length of stay > 2 d	1.035 (0.041*)	**1.035–1.035**	1.113 (< 0.001***)	**1.113–1.113**

The same analysis as in Table 3 was repeated, however, only for patients > 65 years old. Furthermore, patients were excluded from this analysis if they were: (1) male with pre-operative Cr < 0.6, or (2) female with Cr < 0.8 to adjust for the effects of frail or malnourished patients

## Discussion

Preoperative dehydration is a modifiable risk factor that is often overlooked when planning for TKA. Risk stratification and adequate fluid resuscitation may not only help reduce inpatient costs and unexpected medical complications, but also help guide interdisciplinary planning with patients on expected discharge destination [6–8]. With the emphasis on early discharge pathways and value-based healthcare bundled payments, it is important for surgeons to adequately resuscitate dehydrated patients prior to elective TKA to facilitate faster recovery, reduce LOS, and prevent exacerbation of underlying medical comorbidities [2]. Especially in elderly patients with reduced total body water content, dehydration coupled with surgical stress may cause orthostatic hypotension and fatigue further limiting active rehabilitation and return to functional independence [9]. Although dehydration is preventable, the clinical significance of BUN/Cr remains under-recognized in a preoperative setting and should be prioritized as a sensitive marker for predicting complications [10].

Dehydration is one of the most commonly reported diagnoses for Medicare hospital admissions, and nearly 47% of patients undergoing TKA in our study were dehydrated [11]. Increased age was associated with increased severity and prevalence of preoperative dehydration, which may be due to age-related pathophysiologic changes that alter normal regulatory pathways of fluid and electrolyte imbalances [12]. Dehydrated patients, especially elderly individuals, were more likely to acquire a UTI during the acute postoperative period compared to non-dehydrated patients. UTIs represent 13% of all healthcare associated infections in the United States, and in the postoperative period have been previously shown to be linked to complications such as implant failure and revision procedures [13]. Poor adequate fluid intake may increase the risk of UTIs and lead to confusion, falls, and acute kidney injury in susceptible elderly patients [14]. In our study, dehydrated elderly patients had a nearly 30% increased risk of acquiring a UTI compared to hydrated patients. It is important for clinicians to counsel patients on adequate hydration before surgery to prevent prolonged and costly hospital stays [15].

Not only does preoperative volume depletion increase the risk of acquiring a postoperative UTI, but severely dehydrated elderly patients had a > 65% increased risk of developing progressive renal insufficiency during the postoperative period. Renal insufficiency has been previously shown in the arthroplasty literature to increase hospital stay, morbidity, mortality, readmission, and cost [16]. Low circulatory pressures in combination with blood loss-related anemia may lead to reduced renal blood flow and subsequent renal injury [17]. Preventive strategies and patient awareness are the only effective measures to reduce morbidity in cases of postoperative renal dysfunction.

Venous thromboembolism (VTE) remains one of the most common complications and reasons for unplanned readmission among TKA [18]. In our study, severely dehydrated patients had an increased risk of acquiring DVT. The role of dehydration and VTE has been studied in relation to the coagulation system’s increased tendency to form clots when not sufficiently resuscitated [19]. Dehydration may increase the risk of developing VTE, which increases the likelihood of a prolonged hospital stay and healthcare expenditure [20]. In fact, dehydrated patients were more likely to require inpatient hospitalization greater than 2 days and be discharged to a rehabilitation or skilled nursing facility compared to euvolemic patients. The extended stay between dehydration status is important to consider from a billing, hospital bed space, and hospital quality metrics perspective. Fluid optimization of elderly patients may decrease instances of orthostatic hypotension, dizziness, and fatigue that may preclude patients from working with physical therapy and prolong hospitalization.

Not only did dehydrated patients require a greater LOS, but they also had an increased risk for requiring postoperative transfusions. In our study, severely dehydrated elderly patients had a nearly 30% increased association with postoperative transfusion requirement. Several studies have shown an increased risk of infections, costs, and mortality with postoperative allogenic blood transfusions [21]. It is important for healthcare providers to be aware that the concentration of hemoglobin can fluctuate widely with varying levels of intravascular volume within a short period of time [22]. Patients may present with deceivingly normal-high hemoglobin levels due to hemoconcentration that may be masking an underlying pre-existing anemia. During surgery, anesthetic agents cause further hypotension that requires administration of crystalloid fluids. These fluids may then reverse the effects of hemoconcentration, which may uncover postoperative anemia requiring transfusion [22]. This phenomenon may explain the sometimes larger-than-expected decrease in hemoglobin levels after TKA despite minimal blood losses reported intraoperatively. BUN/Cr > 25 may help guide practitioners to identify patients with normal-high reported hemoglobin levels but underlying anemia who may need further iron or erythropoietin treatment prior to surgery.

Overall, while it is difficult to identify at-risk dehydrated patients based on clinical symptoms, previous studies have found BUN/Cr as a reliable laboratory indicator for dehydration [23]. Although BUN/Cr > 20 has been used as an accepted metric for identification of dehydration, further geriatric research has shown the utility of ratios > 25 to indicate severe dehydration [24–27]. This study is unique in that a sub-group analysis of elderly patients was performed to account for age-related pathophysiological changes in the handling of fluid and electrolytes. In order to identify dehydration rather than renal function as a risk factor, serum creatinine levels greater than 1.3 mg/dL were excluded, as levels higher than 1.3 in both genders have been shown to be associated with a reduction of glomerular filtration rate and renal dysfunction [28]. Since Cr levels may be abnormal due to malnutrition in the elderly, this study excluded low Cr level elderly patients who may have false high BUN/Cr ratios not attributable to dehydration.

While this study included a large number of patients, there are limitations to consider when using a national database, including selection bias. Since the data is comprised of a heterogeneous population nationwide, the wide variety of surgeon protocols and expertise may confound outcomes. Although various institutions may implement different preoperative pathways for joint arthroplasty, the inclusion of patients from both academic and private practice settings in rural and urban centers reflect the generalizability of our results. Despite the lack of urinary indices available to help confirm dehydration in our patients, previous literature has shown urine color, specific gravity, and osmolality to have minimal diagnostic value in elderly patients due to age-related changes in renal function and urinary concentration [23]. Finally, lack of information regarding concurrent nephrotoxic medications, such as anti-inflammatories, may confound BUN/Cr and cause values not specifically attributable to dehydration.

## Conclusion

Overall, BUN/Cr is an important preoperative diagnostic tool to identify at-risk dehydrated patients undergoing elective TKA. Dehydration is often an overlooked modifiable risk factor that should be optimized prior to elective TKA to reduce risk of postoperative UTI, DVT, postoperative transfusion, non-home discharge, and increased LOS. As the landscape of healthcare economics shifts to value hospital quality metrics and patient satisfaction outcomes, dehydration status is important to consider as part of a stratified pathway protocol for improved perioperative care.

## Supplementary Information

**Additional file 1: Supplemental Table 1.** Complications based on dehydration level.

## Data Availability

The datasets used and/or analyzed during the current study available from the corresponding author on reasonable request.

## Abbreviations

TKA: Total Knee Arthroplasty
NSQIP: National Surgical Quality Improvement Program
BUN: Blood Urea Nitrogen
BUN/Cr: Blood Urea Nitrogen to Creatinine ratio
DVT: Deep Vein Thrombosis
LOS: Length of Stay
UTI: Urinary Tract Infection
OR: Odds Ratio
CI: Confidence Interval
BMI: Body Mass Index
GLM: General Linear Model
ASA: American Society of Anesthesiologists
VTE: Venous Thromboembolism
