# Supplementary material for: The impact of dehydration on short-term postoperative complications in total knee arthroplasty
Source: BMC Musculoskelet Disord. 2023 Jan 7;24:15. doi: 10.1186/s12891-022-06118-7 (PMC9825029; doi:10.1186/s12891-022-06118-7)
Supplement: Supplementary file 1 — Additional file 1: Supplemental Table 1. Complications based on dehydration level. [file 12891_2022_6118_MOESM1_ESM.docx]

|  | | **Supplemental Table I. Complications based on dehydration level** | | | | | | | | | | | |
| --- | --- | --- | --- | --- | --- | --- | --- | --- | --- | --- | --- | --- | --- |
|  |  | Non-dehydrated | | | | Moderately dehydrated | | | | Severely dehydrated | | | |
|  |  | Cohort | | | | Cohort | | | | Cohort | | | |
|  |  | All patients | | Elder subgroup | | All patients | | Elder subgroup | | All patients | | Elder subgroup | |
|  |  | Count | % | Count | % | Count | % | Count | % | Count | % | Count | % |
| Not discharged home | No | 145259 | 78.92% | 74882 | 74.59% | 69135 | 76.68% | 41756 | 72.76% | 52010 | 73.92% | 32943 | 69.65% |
|  | Yes | 38796 | 21.08% | 25506 | 25.41% | 21028 | 23.32% | 15636 | 27.24% | 18349 | 26.08% | 14352 | 30.35% |
| Infection | No | 182020 | 98.89% | 99379 | 98.99% | 89221 | 98.96% | 56792 | 98.95% | 69702 | 99.07% | 46861 | 99.08% |
|  | Yes | 2035 | 1.11% | 1009 | 1.01% | 942 | 1.04% | 600 | 1.05% | 657 | 0.93% | 434 | 0.92% |
| Cardiac Arrest or MI | No | 183651 | 99.78% | 100098 | 99.71% | 89941 | 99.75% | 57203 | 99.67% | 70185 | 99.75% | 47147 | 99.69% |
|  | Yes | 404 | 0.22% | 290 | 0.29% | 222 | 0.25% | 189 | 0.33% | 174 | 0.25% | 148 | 0.31% |
| Wound Disruption | No | 183665 | 99.79% | 100203 | 99.82% | 89989 | 99.81% | 57269 | 99.79% | 70224 | 99.81% | 47208 | 99.82% |
|  | Yes | 390 | 0.21% | 185 | 0.18% | 174 | 0.19% | 123 | 0.21% | 135 | 0.19% | 87 | 0.18% |
| Pneumonia | No | 183534 | 99.72% | 100071 | 99.68% | 89926 | 99.74% | 57209 | 99.68% | 70155 | 99.71% | 47133 | 99.66% |
|  | Yes | 521 | 0.28% | 317 | 0.32% | 237 | 0.26% | 183 | 0.32% | 204 | 0.29% | 162 | 0.34% |
| Unplanned Intubation | No | 183836 | 99.88% | 100238 | 99.85% | 90043 | 99.87% | 57302 | 99.84% | 70273 | 99.88% | 47231 | 99.86% |
|  | Yes | 219 | 0.12% | 150 | 0.15% | 120 | 0.13% | 90 | 0.16% | 86 | 0.12% | 64 | 0.14% |
| Pulmonary Embolism | No | 183089 | 99.48% | 99803 | 99.42% | 89680 | 99.46% | 57038 | 99.38% | 69971 | 99.45% | 47006 | 99.39% |
|  | Yes | 966 | 0.52% | 585 | 0.58% | 483 | 0.54% | 354 | 0.62% | 388 | 0.55% | 289 | 0.61% |
| On Ventilator > 48 h | No | 183957 | 99.95% | 100317 | 99.93% | 90111 | 99.94% | 57349 | 99.93% | 70326 | 99.95% | 47271 | 99.95% |
|  | Yes | 98 | 0.05% | 71 | 0.07% | 52 | 0.06% | 43 | 0.07% | 33 | 0.05% | 24 | 0.05% |
| Progressive Renal Insufficiency | No | 183904 | 99.92% | 100311 | 99.92% | 90084 | 99.91% | 57348 | 99.92% | 70301 | 99.92% | 47249 | 99.90% |
|  | Yes | 151 | 0.08% | 77 | 0.08% | 79 | 0.09% | 44 | 0.08% | 58 | 0.08% | 46 | 0.10% |
| Acute Renal Failure | No | 183979 | 99.96% | 100338 | 99.95% | 90139 | 99.97% | 57374 | 99.97% | 70339 | 99.97% | 47280 | 99.97% |
|  | Yes | 76 | 0.04% | 50 | 0.05% | 24 | 0.03% | 18 | 0.03% | 20 | 0.03% | 15 | 0.03% |
| Urinary Tract Infection | No | 182882 | 99.36% | 99573 | 99.19% | 89376 | 99.13% | 56806 | 98.98% | 69736 | 99.11% | 46785 | 98.92% |
|  | Yes | 1173 | 0.64% | 815 | 0.81% | 787 | 0.87% | 586 | 1.02% | 623 | 0.89% | 510 | 1.08% |
| Stroke CVA | No | 183928 | 99.93% | 100283 | 99.90% | 90087 | 99.92% | 57327 | 99.89% | 70297 | 99.91% | 47241 | 99.89% |
|  | Yes | 127 | 0.07% | 105 | 0.10% | 76 | 0.08% | 65 | 0.11% | 62 | 0.09% | 54 | 0.11% |
| Severe bleeding requiring transfusion | No | 177492 | 96.43% | 96423 | 96.05% | 86723 | 96.18% | 54994 | 95.82% | 67236 | 95.56% | 45001 | 95.15% |
|  | Yes | 6563 | 3.57% | 3965 | 3.95% | 3440 | 3.82% | 2398 | 4.18% | 3123 | 4.44% | 2294 | 4.85% |
| DVT | No | 182650 | 99.24% | 99610 | 99.23% | 89480 | 99.24% | 56922 | 99.18% | 69784 | 99.18% | 46865 | 99.09% |
|  | Yes | 1405 | 0.76% | 778 | 0.77% | 683 | 0.76% | 470 | 0.82% | 575 | 0.82% | 430 | 0.91% |
| Sepsis | No | 183688 | 99.80% | 100189 | 99.80% | 90006 | 99.83% | 57277 | 99.80% | 70250 | 99.85% | 47215 | 99.83% |
|  | Yes | 367 | 0.20% | 199 | 0.20% | 157 | 0.17% | 115 | 0.20% | 109 | 0.15% | 80 | 0.17% |
| Septic Shock | No | 183973 | 99.96% | 100325 | 99.94% | 90111 | 99.94% | 57356 | 99.94% | 70327 | 99.95% | 47266 | 99.94% |
|  | Yes | 82 | 0.04% | 63 | 0.06% | 52 | 0.06% | 36 | 0.06% | 32 | 0.05% | 29 | 0.06% |
| C. diff | No | 121949 | 99.92% | 67698 | 99.89% | 59913 | 99.92% | 38672 | 99.90% | 45980 | 99.93% | 31137 | 99.93% |
|  | Yes | 103 | 0.08% | 74 | 0.11% | 46 | 0.08% | 37 | 0.10% | 30 | 0.07% | 23 | 0.07% |
| Periprosthetic fracture | No | 183639 | 99.77% | 100170 | 99.78% | 89993 | 99.81% | 57273 | 99.79% | 70204 | 99.78% | 47183 | 99.76% |
|  | Yes | 416 | 0.23% | 218 | 0.22% | 170 | 0.19% | 119 | 0.21% | 155 | 0.22% | 112 | 0.24% |
| LOS > 2 d | No | 102898 | 55.91% | 53619 | 53.41% | 48923 | 54.26% | 29683 | 51.72% | 36267 | 51.55% | 23041 | 48.72% |
|  | Yes | 81157 | 44.09% | 46769 | 46.59% | 41240 | 45.74% | 27709 | 48.28% | 34092 | 48.45% | 24254 | 51.28% |

**Supplemental Table I. Occurrence of complications after total knee arthroplasty.**

The table shows the raw counts of complications stratified by dehydration level and subgroup (all patients vs elder subgroup ≥ 65 y).
